# Association between Growth Differentiation Factor-15 (GDF-15) Serum Levels, Anorexia and Low Muscle Mass among Cancer Patients

**DOI:** 10.3390/cancers13010099

**Published:** 2020-12-31

**Authors:** Alessio Molfino, Maria Ida Amabile, Giovanni Imbimbo, Veronica Rizzo, Federica Pediconi, Carlo Catalano, Alessandra Emiliani, Roberta Belli, Cesarina Ramaccini, Claudia Parisi, Giuseppe Nigri, Maurizio Muscaritoli

**Affiliations:** 1 Department of Translational and Precision Medicine, Sapienza University of Rome, 00185 Rome, Italy; imbimbo.1638090@studenti.uniroma1.it (G.I.); alessandra.emiliani1@gmail.com (A.E.); roberta.belli@uniroma1.it (R.B.); cesarina.ramaccini@uniroma1.it (C.R.); maurizio.muscaritoli@uniroma1.it (M.M.); 2 Department of Surgical Sciences, Sapienza University of Rome, 00185 Rome, Italy; mariaida.amabile@uniroma1.it; 3 Department of Radiological, Oncological and Anatomopathological Sciences, Sapienza University of Rome, 00185 Rome, Italy; veronica.rizzo@uniroma1.it (V.R.); federica.pediconi@uniroma1.it (F.P.); carlo.catalano@uniroma1.it (C.C.); 4 Department of Medical and Surgical Sciences and Translational Medicine, Sapienza University of Rome, 00185 Rome, Italy; parisiclaudia168@gmail.com (C.P.); giuseppe.nigri@uniroma1.it (G.N.)

**Keywords:** cancer, anorexia, tumor-secreted factors, GDF-15, muscularity, skeletal muscle, body weight loss, wasting

## Abstract

**Simple Summary:**

In our study, the novel inflammatory cytokine Growth Differentiation Factor-15 (GDF-15) has been found elevated in patients with gastrointestinal and lung cancer and associated with anorexia. Patients with gastrointestinal cancer were found more anorexic (based on the FAACT score) and showed higher GDF-15 serum levels than patients with lung cancer. We also evaluated the muscularity status of the patients by CT scan. No difference was found in GDF-15 levels between cancer patients with low muscle mass vs. those with moderate/high muscularity and between patients with body weight loss vs. those with stable weight. Based on our observations, we confirm the role of GDF-15 in the pathogenesis of anorexia in cancer, although the mechanisms of action of this cytokine in cancer should be further unveiled also regarding its potential involvement in changes in muscularity.

**Abstract:**

The pathophysiology of cancer anorexia is complex and serum biomarkers, including growth and differentiation factor(s) (GDF), may be modulated. We explored the association(s) between GDF-15 serum levels and anorexia and, secondarily, with low muscle mass and body weight loss in cancer patients. We considered gastrointestinal and lung cancer patients (CP) and healthy BMI-matched controls. The FAACT-questionnaire was administered to diagnose anorexia and we calculated the L3-SMI by CT scan to assess low muscularity, setting their cutoff values at the lowest tertile. GDF-15 serum levels were assessed by ELISA. We enrolled 59 CP and 30 controls; among CP, 25 were affected by gastrointestinal and 34 by lung cancer. Anorexia was present in 36% of CP. Gastrointestinal CP resulted more anorexic compared to lung CP (*p* = 0.0067). Low muscle mass was present in 33.9% of CP and L3-SMI was lower in gastrointestinal compared to lung CP (*p =* 0.049). The GDF-15 levels were higher in CP vs. controls (*p* = 0.00016), as well as in anorexic vs. non-anorexic CP (*p* = 0.005) and vs. controls (*p* < 0.0001). Gastrointestinal CP showed higher GDF-15 levels vs. lung CP (*p* = 0.0004). No difference was found in GDF-15 between CP with low muscle mass and those with moderate/high muscularity and between patients with body weight loss and those with stable weight. Our data support the involvement of GDF-15 in the pathogenesis of cancer anorexia. The mechanisms of action of GDF-15 in cancer should be further clarified also regarding the changes in muscularity.

## 1. Introduction

During cancer, several metabolic changes have been documented due to multiple and often unclear mechanisms [1]. In particular, energy metabolism is deeply altered in cancer leading to involuntary decrease in body weight mainly due to appetite loss (i.e., anorexia) and muscle mass derangements [1].

Anorexia is a clinically relevant problem in chronic diseases, including cancer, and is associated with poor outcomes, such as decreased survival [2]. In particular, in a miscellaneous cohort of cancer patients, the prevalence of anorexia was reported to account for 41% [3].

Several factors were proposed to be involved in the pathogenesis of cancer-associated appetite loss, including increased pro-inflammatory cytokines, tumor-derived catabolic factors, anticancer treatments and changes in gut microbiota [4,5,6]. Moreover, the pathophysiology of cancer anorexia seems to be characterized by the overcoming of anorexic signals with respect to the orexigenic acting at hypothalamic level [7,8].

A novel inflammatory cytokine, the Growth Differentiation Factor-15 (GDF-15), a member of the transforming growth factor beta family, has been found significantly elevated in cancer patients [4]. This cytokine (named also as MIC-1, PLAB, NAG1, or PTGFB) was recently investigated for obesity treatment and authors suggested to consider GDF-15 a member of Glial Cell-Derived Neurotrophic Factor (GDNF) family due to the high affinity with GDNF family receptor α-like (GFRAL) and its co-receptor Ret proto-oncogene (RET) [9]. Experimental data indicated that GDF-15 may play a key role in the pathogenesis of cancer-related anorexia [10] and therefore potentially implicated in determining protein-energy malnutrition [11]. Borner et al. found that GDF-15 induced sickness behavior as result of its action in lowering food intake and, in an experimental model, GDF-15 release was enhanced by chemotherapy [12]. On the other hand, the blockade of GDF-15 action might ameliorate gastrointestinal symptoms (e.g., vomiting and/or nausea) during chemotherapy [12]. However, no conclusive data are available in humans on the role of GDF-15 in cancer-associated anorexia.

For this reason, we aimed to assess (i) primarily the association between GDF-15 circulating levels and anorexia and (ii) secondarily with low muscle mass or body weight loss in cancer patients naïve to anti-cancer treatments.

## 2. Results

### 2.1. Patient’s Characteristics

We enrolled a total of 59 cancer patients (66% male) and 30 subjects serving as controls (43% male). Among the total of cancer patients, 25 were affected by gastrointestinal cancer (9 pancreatic, 7 gastric, and 9 colorectal cancer patients), enrolled at the Department of Medical-Surgical Sciences and Translational Medicine, Sapienza University of Rome, and 34 were lung cancer patients (33 with NSCLC and 1 with SCLC), enrolled at Department of Translational and Precision Medicine (formerly Department of Clinical Medicine) and at the Division of Oncology, Sapienza University of Rome. None of the gastrointestinal and lung cancer patients received anti-cancer treatments before the enrollment. Patient’s characteristics are shown in Table 1. The most common comorbidities were hypertension (47%), dyslipidemia (19%), and diabetes (17%). The mean body mass index (BMI) was 25.19 ± 3.94 kg/m^2^ and the body weight loss in the previous six months was present in 49 out of 59 patients (83%) (Table 1).

The control group included 15 subjects, enrolled at the Department of Medical-Surgical Sciences and Translational Medicine, Sapienza University of Rome, before undergoing abdominal surgery for non-neoplastic diseases (e.g., inguinal hernia, abdominal wall surgery) and additional 15 healthy volunteers enrolled at the Department of Translational and Precision Medicine, Sapienza University of Rome, for a total of 30 subjects (43% male). The control group showed a median age of 61 years (53.25; 65.5) and a BMI (kg/m^2^) of 26.51 ± 4.46; subjects under treatment for hypertension were 15 (50%) (Table 1).

Based on the Functional Assessment of Anorexia/Cachexia Therapy (FAACT) score lowest tertile, anorexia was present in 21 out of 59 cancer patients (Table 1), showing a mean FAACT score of 17.3 ± 4.6, whereas patients with no anorexia (38/59) showed a FAACT score of 31.9 ± 4.7 (*p* < 0.0001). Gastrointestinal cancer patients showed a FAACT score of 23.3 ± 6.9 resulting significantly lower with respect to lung cancer patients (29.2 ± 8.7) (*p* = 0.0067). No differences were observed between males and females in terms of FAACT score (*p* = 0.128).

Based on the lowest sex-specific tertile of third lumbar vertebrae (L3)–Skeletal Muscle Index (SMI) (cm^2^/m^2^), low muscle mass was defined with the cut-offs of <35.2 for women and <44.92 for men. The L3-SMI values according to sex is shown in Table 1. Among our entire cohort, patients with low muscle mass were 20 out of 59 patients (33.9%). The mean L3-SMI was lower in gastrointestinal cancer patients compared to patients with lung cancer (41.42 ± 8.62 vs. 46.03 ± 8.75) (*p* = 0.049). No differences were observed in terms of prevalence of low muscle mass between anorexic and non-anorexic cancer patients (*p* = 0.427).

### 2.2. GDF-15 Serum Levels in Anorexic Cancer Patients, Non-Anorexic Cancer Patients and in Controls

**Figure 1:**
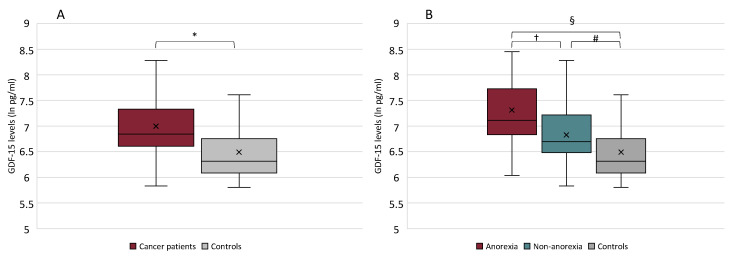
Growth Differentiation Factor 15 (GDF-15) serum levels in cancer patients vs. controls (* *p* = 0.00016) (**A**) and in cancer patients with anorexia, without anorexia, and in controls (Kruskal–Wallis test between the three groups *p* = 0.00004) (**B**). † *p* = 0.005; § *p* < 0.0001; # *p* = 0.006.

The GDF-15 serum levels (pg/mL) were significantly higher in cancer patients (median 6.84, IQR 6.61; 7.32) with respect to controls (median 6.31, IQR 6.09; 6.73) (*p* = 0.00016) (Figure 1A).

Based on the presence/absence of anorexia, the GDF-15 serum levels were significantly higher in anorexic (median 7.11, IQR 6.86;7.49) vs. non-anorexic cancer patients (median 6.70 IQR 6.50; 7.16) (*p* = 0.005) and vs. controls (median 6.31 IQR 6.09; 6.73) (*p* < 0.0001), as well as higher in non-anorexic cancer patients vs. controls (*p* = 0.006) (Figure 1B).

**Figure 2:**
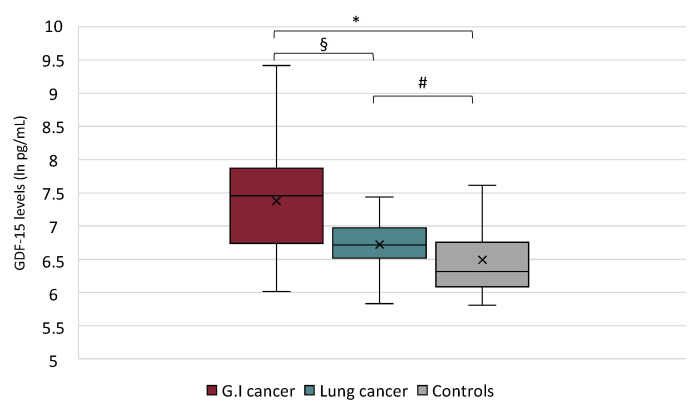
Growth Differentiation Factor 15 (GDF-15) serum levels in gastrointestinal (G.I.) vs. lung cancer patients and vs. controls (Kruskal–Wallis test *p* < 0.0001); § *p* = 0.0004; * *p* = 0.00006; # *p* = 0.008.

Gastrointestinal cancer patients showed higher GDF-15 serum levels (median of 7.46 IQR, 6.80; 7.77) with respect to lung cancer patients (median 6.72 IQR 6.54; 6.95) (*p* = 0.0004). Both cancer groups showed GDF-15 serum levels higher compared to controls (*p* = 0.00006; *p* = 0.008, respectively) (Figure 2).

**Figure 3:**
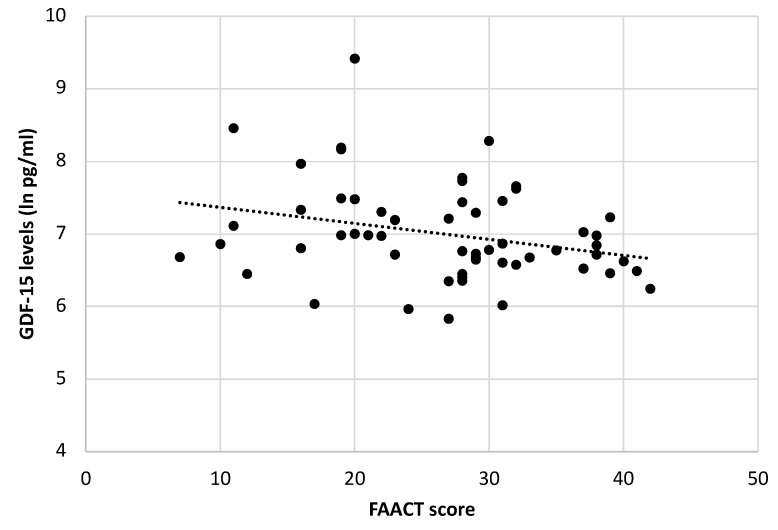
Correlation between the Functional Assessment of Anorexia/Cachexia Therapy (FAACT) score and GDF-15 serum levels (ln pg/mL) (r = −0.280; *p* = 0.03).

Moreover, among all cancer patients, we observed a negative correlation between GDF-15 serum levels and FAACT score (r = −0.280, *p* = 0.03) (Figure 3). No significant differences were seen in GDF-15 levels according to age and sex in both cancer groups and controls.

### 2.3. GDF-15 Serum Levels in Cancer Patients with Low Muscle Mass vs. Moderate/High Muscle Mass and in Controls

**Figure 4:**
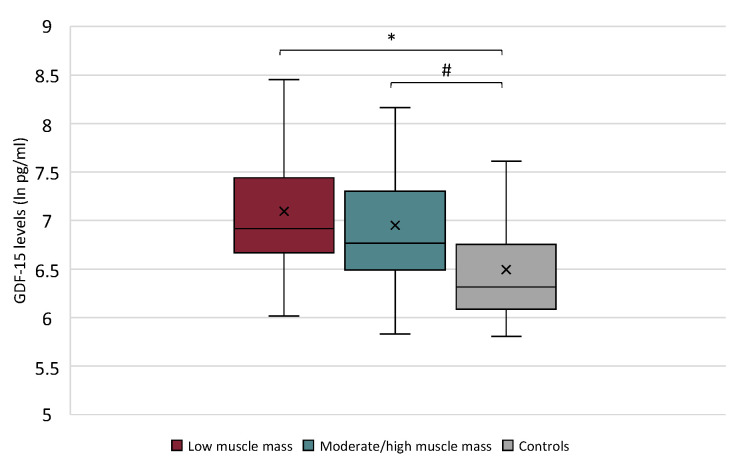
**Growth Differentiation Factor 15 (**GDF-15) serum levels in cancer patients with low muscle mass vs. those with moderate/high muscle mass and vs. controls (Kruskal–Wallis test *p* = 0.0005). * *p* = 0.0006; # *p* = 0.0017.

The GDF-15 serum levels were significantly higher in patients with low muscle mass (median 6.92, IQR 6.70; 7.37) with respect to controls (*p* = 0.0006), as well as higher in patients with moderate/high muscle mass (median 6.76, IQR 6.55; 7.27) compared to control group (*p* = 0.0017). However, no differences were found in GDF-15 serum levels between patients with low muscle mass and those with moderate/high muscularity (*p* = 0.313) (Figure 4). Among all cancer participants, no correlation was found between GDF-15 serum levels and L3-SMI (r = −0.168, *p* = 0.203).

### 2.4. Association between Body Weight Loss, Anorexia, and GDF-15 Serum Levels

Among both cancer groups, the median body weight loss was 6.48% (IQR 4.08; 9.30).

No association between body weight loss and anorexia was present among cancer patients (*p* = 0.06).

Lung cancer patients showed a higher median body weight loss (%) with respect to gastrointestinal cancer patients (7.72 vs. 4.23) (*p* = 0.008). None of the subjects in the control group showed body weight loss in the previous six months (Table 1). The median GDF-15 circulating levels were not different between cancer patients with body weight loss vs. patients with stable body weight (6.86 vs. 6.68) (*p* = 0.258). Finally, no correlation was detected between GDF-15 serum levels and percent of body weight loss (r = −0.210, *p* = 0.148).

## 3. Discussion

By our study design, we aimed to ascertain the association of the novel cytokine GDF-15 and anorexia during cancer and its potential relationship with low muscle mass and body weight loss, which are well known hallmarks of deranged nutritional and metabolic status in cancer. Interestingly, the GDF-15 serum levels were significantly higher in patients with cancer compared to our control group and, more importantly, anorexic cancer patients showed higher GDF-15 concentrations compared to those without anorexia. In particular, low appetite was found in 36% (21/59) of patients with gastrointestinal and lung cancer, similarly to studies conducted among other cohorts of lung and gastrointestinal cancer patients where the prevalence of anorexia ranged 13–55% [13,14].

In this light, the tool we used to discriminate the presence of anorexia (the lowest FAACT score tertile) appears reliable and comparable to the one used in a similar setting (FAACT ≤ 24) [13], although a validated FAACT cutoff value is not available. In particular, appetite was significantly lower in patients with gastrointestinal cancer compared to those with lung cancer. These results are in line with previous findings obtained among patients at their first medical oncology visit, indicating a high prevalence of anorexia and malnutrition in gastroesophageal and pancreatic cancer patients, even at an initial stage of the disease [3]. Moreover, it has to be considered that, in our cohort, lung cancer patients presented a more advanced stage of the disease with respect to gastrointestinal cancer patients and this may justify the greater body weight loss observed in the lung group, likely due to different underlying catabolic mechanisms beyond anorexia.

Our previous data confirmed the clinical relevance of anorexia also in lung cancer patients, suggesting a dysregulated control of appetite in this setting at least in part associated with changes in gastrointestinal peptides and cytokines [15].

The role of GDF-15 in the pathogenesis of cancer anorexia was initially described by Johnen et al. demonstrating in mouse models a satietogenic effect of GDF-15 by central hypothalamic activation [11]. Moreover, Yang et al. further elucidated the GDF-15 anorexigenic pathway showing that GDF-15 binds the GFRAL and its coreceptor RET within the area postrema and nucleus of the solitary tract inducing low appetite and the consequent reduction in food intake [9].

Lerner et al., studying the effects of GDF-15 in humans, revealed an association between this cytokine and body weight loss but not with low appetite assessed by the Anderson Symptom Assessment Scale [10]. In our study, we used the FAACT questionnaire to assess the presence and the grade of anorexia, which may be more comprehensive when compared to other appetite tools, including the Anderson Symptom Assessment Scale, due to the inclusion of different domains, such as taste and smell alterations, nausea, and vomiting, which are symptoms that may all characterize the sickness behavior of patients with cancer [16]. Interestingly, authors confirmed that GDF-15 determines anorexia by inducing nausea and vomiting in mammalian models [12].

Noteworthy, gastrointestinal cancer patients, who were characterized by a higher grade of anorexia (considering their lower FAACT score), showed higher GDF-15 serum levels compared to lung cancer patients that may be, at least in part, related to the heterogeneity of the gastrointestinal group. This observation, although to be further confirmed, suggests an interesting relationship between a higher grade of anorexia and increased GDF-15 circulating levels. In this light, our results indicate a negative correlation, although weak, between FAACT score and GDF-15 levels in our cohort of cancer patients.

Anorexia is clinically important considering that it represents one of the main causes of nutritional derangements in cancer [16], including the loss of muscle mass [17]. Therefore, we hypothesize that GDF-15 could be implicated in the development of low muscle mass and body weight loss in cancer patients. Low body weight with specific losses of muscle mass and adiposity represents hallmark of cancer-related cachexia, a disorder characterized by a combination of metabolic alterations and low food intake [4]. The clinical picture of cancer-associated cachexia may vary according to the type and stage of cancer, and therefore it may reveal with different severity. Moreover, cancer cachexia involves several mediators produced from the cancer cells and the tumor microenvironment, i.e., pro-inflammatory mediators including GDF-15 [4]. The data obtained in experimental models documented that GDF15-mediated effects (i.e., low food intake and body weight loss) observed in obese mice were lost by the GFRAL-knockout [9]. In addition, GFRAL was not expressed in peripheral tissues, supporting that energy homeostasis was centrally regulated [9]. Moreover, GFRAL knockout animals presented an increased food intake under stressed conditions and were resistant to chemotherapy-related loss of appetite and of body weight [18].

During cancer, GDF-15 could activate these “non-homeostatic” pathways–specifically acting on the GFRAL-expressing neurons localized in the area postrema and nucleus of the solitary tract—which are able to regulate body weight [18]. In particular, Suriben et al. investigated the effects of an antagonistic monoclonal antibody (named 3P10), targeted to inhibit GDF-15 interaction with its receptor and coreceptor, and documented that this antibody successfully prevented GDF15-induced anorexia and, in turn, muscle loss via neuroendocrine pathways in animals [19].

By our results, we did not find changes in GDF-15 circulating levels among patients with low muscle mass vs. moderate-high muscle mass evaluated by CT scan, as well as in patients with body weight loss compared to those with a stable weight. Although different experimental studies converge on the role of GDF-15 as pivotal in determining anorexia and body weight loss, the evidence in humans is limited and apparently controversial. In fact, GDF-15 was previously observed to be related with inflammatory markers in gastrointestinal cancer patients but not with nutritional parameters, such as body weight loss, anthropometry, and only weakly with low dietary food intake [20]. On the other hand, the GDF-15 circulating levels were found higher in cancer patients with body weight loss with respect to those with a stable weight [10], observations that were also recently confirmed in experimental models [21]. These different results obtained by others, including patients suffering by a miscellaneous of cancer diseases (only male) [10], or by esophago-gastric cancer [20] and by our study—focused on lung and gastrointestinal cancer patients—may be in part related to the different populations enrolled and likely to a different stage of cancer disease.

We acknowledge the limitations of our study. Our findings were in part limited by the study design (cross-sectional), which did not evaluate anorexia, low muscle mass, and body weight loss prospectively according to changes in GDF-15 levels overtime, and therefore a longitudinal analysis of our data may add important information on this aspect. Although we have collected data from two different cohorts of patients (gastrointestinal and lung cancer), the study of the GDF-15 serum levels in additional different cancer type and stages might reveal whether anorexia is present/absent according to the GFD-15 serum levels or to the type/stage of the underlying cancer disease. We did not focus on changes in adiposity, which were considered relevant in determining malnutrition and cachexia, especially by the activation of the adipose triglyceride lipase in white adipose tissue in experimental models of cancer cachexia [19].

## 4. Materials and Methods

### 4.1. Study Design and Participants

This was an observational, two-center, controlled study performed on cancer patients enrolled at the Department of Translational and Precision Medicine and Department of Surgical Sciences, Sapienza University of Rome, Italy. The study was performed in accordance to the Declaration of Helsinki and approved by the local Ethical Committees (Sapienza University of Rome, Azienda Policlinico Umberto I - prot. N. 1162/17, and Sant’Andrea Hospital - prot. N. 167SA_2017, Rome, Italy). We considered lung and gastrointestinal cancer patients at their first oncology visit; for lung cancer we considered either non-small-cell lung carcinoma and small-cell lung carcinoma, and for the gastrointestinal tract, either gastric, colorectal, and pancreatic cancer, undergoing surgery for tumor resection. Inclusion criteria included age ≥ 18 years and the ability to provide informed consent. We excluded patients with chronic disease related-malnutrition different by cancer, such as chronic kidney disease, chronic heart failure, liver cirrhosis, as well as patients with cognitive impairment, chronic infections, dysphagia, or occlusion of the gastrointestinal tract.

### 4.2. Nutritional and Clinical Assessment

We collected data on nutritional status, i.e., usual weight and involuntary body weight loss in the previous 6 months by patient’s self-report, and we measured current weight, height, and calculated BMI during the study visit.

During the visit, in a fasting condition, we collected blood samples in EDTA tubes and centrifugated to analyze serum biomarkers such as albumin and C-reactive protein with standard automated techniques. We also assessed hemoglobin levels among all the participants.

Moreover, from patient clinical records, we collected data on the staging and histology of the cancer and on patient’s medical history, including comorbidities.

### 4.3. Anorexia Assessment

Anorexia was assessed interviewing patients during the first oncology visit by the FAACT questionnaire, which was endorsed by the European Society for Clinical Nutrition and Metabolism [22]. FAACT score consists of 12 questions investigating different domains of anorexia, such as appetite, low food intake, presence of nausea or vomiting, and each question scores a minimum of 0 (not at all) and a maximum of 4 (very much) based on a Likert scale [22].

Different cut-off values were recently proposed for the diagnosis of anorexia [13,23]. However, these cutoffs were used in large studies aimed at assessing the clinical relevance of anorexia in terms of survival and other clinical outcomes [2,24]. Considering the aim of our study and the relatively limited number of participants included in our cohort, we set our FAACT score at the lowest tertile. This approach allowed comparison between groups with a relatively low FAACT score (anorexic) to be compared to higher FAACT score (non-anorexic).

### 4.4. Evaluation of Muscle Mass by CT Scans

To determine SMI, it was initially necessary to estimate the skeletal muscle area (SMA) that includes psoas, paraspinal muscles (erector spinae and quadratus lumborum), and the abdominal wall muscles (rectus abdominis, transversus abdominis, external oblique, and internal oblique). For each patient, un-enhanced abdominal CT scans were chosen and the axial slices at the level of the third lumbar vertebrae (L3) were identified. All the axial slices were analyzed in a separate workstation, using Osirix Lite DICOM Viewer (version 11.0.3, Geneva, Switzerland), as previously validated [25]. As first step, we identified the outer abdominal fascia and inner muscle wall to create enclosed regions of interest. Muscle cross-sectional areas were identified by setting the threshold range for skeletal muscle (from −29 to +150 Hounsfield Units), and SMI (cm^2^/m^2^) was calculated dividing respective SMA (cm^2^) by each patients’ height-squared (m^2^).

Patients with low muscle mass were identified as those with the lowest sex-specific tertile of SMI, as previously described [26]. This approach was reliable among similar cohorts of cancer patients allowing a balanced distribution between patients with low muscle mass vs. moderate-high muscle mass [26,27]

### 4.5. Serum GDF-15 Levels

Serum GDF-15 concentrations were assessed by the enzyme linked immunosorbent assay kit (Human GDF-15 Elisa Kit, Abcam, Cambridge, UK). Considering the skewed distribution, we transformed the GDF-15 values into the natural logarithm (ln), as previously described [28].

### 4.6. Statistical Analyses

We described patients’ characteristics using mean ± standard deviation and median with 25th and 75th percentiles for continuous normally and non-normally distributed variables, respectively. Normal distribution was evaluated by Shapiro Wilk test. Categorical variables were shown as number (%). We evaluated differences among three groups (anorexic, non-anorexic and controls; low muscle mass, moderate/high muscle mass, and controls) by Analysis of Variance (ANOVA) and by the Kruskal–Wallis test, as appropriate. We also used the Two-tailed t-test or Mann–Whitney, according to normal or non-normal distribution, to evaluate differences between groups. Association between categorical variables were assessed by Chi-square test.

The correlation between serum GDF-15 levels and FAACT score, L3-SMI and body-weight loss were analyzed by Pearson’s test. A *p*-value < 0.05 was considered statistically significant.

## 5. Conclusions

We found increased GDF-15 serum levels in cancer patients, and in particular in those with anorexia when compared to cancer patients with normal appetite. Patients with gastrointestinal cancer resulted in being more anorexic and with higher GDF-15 levels than lung cancer patients. In our setting, no association was detected between GDF-15 and low muscle mass and body weight loss. Additional data are essential to confirm our results, in particular to clarify the potential role of GDF-15 in determining low muscle mass in cancer independently or not by the presence of anorexia.

## Figures and Tables

**Table 1:** Patient’s characteristics.

	Cancer Patients (*N* = 59)		Controls (*N* = 30)	* *p*-Value
Parameter	Lung (*N* = 34)	Gastrointestinal (*N* = 25)		
Male (%)	27 (79%)	12 (48%)	13 (43%)	0.0394
Age (y)	67.97 ± 12.03	71.64 ± 11.22	58.53 ± 11.85	<0.0001
Body mass index, kg/m^2^	23.83 ± 3.91	27.04 ± 3.22	26.51 ± 4.46	0.158
Current weight, kg	68.43 ± 12.96	76.48 ± 12.58	74.5 ± 13.01	0.371
Usual weight, kg	73.82 ± 13.26	81.22 ± 13.34	73.54 ± 11.93	0.312
Hemoglobin, g/dl	13.0 (12.2; 13.4)	12.1 (9.3; 13.0)	13.7 (12.2; 15.5)	0.0108
C-reactive protein, mg/dl	5.85 (2.87; 9.40)	1.42 (0.28; 3.23)	0.29 (0.17; 0.62)	<0.0001
Albumin, g/dl	3.74 (3.35; 4.05)	3.20 (3.00; 3.50)	4 (3.76; 4.1)	0.0003
Presence of anorexia, n (%)	8 (24%)	13 (52%)	/	
Percent of body weight loss	7.72 (5.31; 12.92)	4.23 (3.53; 7.83)	0 (0; 0)	<0.0001
L3-SMI, cm^2^/m^2^	M, 47.60 ± 8.78 F, 39.97 ± 5.78	M, 44.28 ± 8.73 F, 38.77 ± 7.93	/	
Type of cancer				
Gastric, n (%)	/	7 (12%)		
Pancreas, n (%)	/	9 (15%)		
Colorectal, n (%)	/	9 (15%)		
Stage				
I–II	3	15		
III–IV	31	10		

* Cancer patients (n = 59) vs. Controls. Data are shown as Mean ± SD. Median (interquartile range) is shown for non-normally distributed variables. Abbreviations: L3-SMI, skeletal muscle index at L3.

## Data Availability

The data presented in this study are available on request from the corresponding author.
